# Multi-Omics Insights into Microbial Community Dynamics and Functional Shifts During Double-Round Bottom Fermentation of Strong-Flavor Baijiu

**DOI:** 10.3390/foods14244228

**Published:** 2025-12-09

**Authors:** Jiao Li, Yaqi Guo, Yang Yang, Shu Li, Tao Xu, Ruiqi Zeng, Songtao Wang, Caihong Shen, Zhenghong Xu, Yong Zuo, Chen Xiao

**Affiliations:** 1 Key Laboratory of the Evaluation and Monitoring of Southwest Land Resources (Ministry of Education), Sichuan Normal University, Chengdu 610066, China; 2 College of Life Science, Sichuan Normal University, Chengdu 610101, China; 3 Luzhou Pinchuang Technology Co., Ltd., Luzhou 646000, China; 4 National Engineering Research Center of Solid-State Brewing, Luzhou 646000, China; 5 Luzhou Laojiao Co., Ltd., Luzhou 646000, China; 6 Innovation Center for Advanced Brewing Science and Technology, Sichuan University, Chengdu 610065, China

**Keywords:** distilled spirits, violate compounds, microbial community, metabolic pathways reconstruction

## Abstract

Double-round bottom fermentation (DRBF) represents an important technological innovation in strong-flavor Baijiu production, yet the microbial succession and metabolic mechanisms underlying this process remain insufficiently understood. In this study, physicochemical analyses combined with multi-omics approaches were employed to elucidate the dynamic variations in physicochemical parameters, volatile compounds, and microbial community structure and function during DRBF, as well as to reconstruct key metabolic pathways involved in fermentation. A total of 153 volatile compounds were identified, with esters, alcohols, and acids as the major components showing distinct accumulation patterns across fermentation stages. High-throughput sequencing detected 505 bacterial and 175 fungal genera, dominated by *Lactobacillus*, *Aspergillus*, and *Saccharomyces*. Functional annotation revealed that metabolic pathways predominated, shifting from energy- and growth-related processes in the early stage to amino acid, fatty acid, and secondary metabolite biosynthesis in the later stage. Reconstruction of metabolic pathways identified 57 key enzymes linking starch degradation, pyruvate metabolism, the tricarboxylic acid (TCA) cycle, and ester biosynthesis, indicating cooperative metabolism among bacteria, yeasts, and molds. These findings elucidate the synergistic metabolic mechanisms of flavor formation during DRBF and provide a scientific basis for optimizing fermentation control and improving Baijiu quality.

## 1. Introduction

Chinese Baijiu, as one of the world’s oldest distilled spirits, represents a unique fermentation system characterized by complex microbial interactions and distinctive flavor formation mechanisms [1,2]. According to recent industry statistics, large-scale liquor enterprises produced more than 36 billion liters of alcoholic beverages between January and October 2024, with sales revenues approaching 750 billion CNY [3]. Strong-flavor Baijiu is one of the principal aroma types in the Chinese market, characterized by an aroma profile dominated of ethyl hexanoate [1,4,5]. The distinctive quality of strong-flavor Baijiu originates from its open and highly complex brewing microecosystem, which depends on the synergistic solid-state fermentation of multiple microbial species [6]. Within this system, the brewing environment and the metabolic activity of microbiota play important roles in shaping the flavor profile of Baijiu [7,8,9].

The production of strong-flavor Baijiu involves a series of techniques [10], among which the “double-round bottom fermentation” (DRBF) (also called “Shuanglundi fermentation” in Chinese) process represents an important technological innovation developed to enhance the quality of base liquor [11]. In this process, the fermented grains at the pit bottom undergo an additional fermentation cycle, characterized by the introduction of a proper proportion of Daqu (a traditional Baijiu fermentation starter), yellow water (a primary liquid by-product of the strong-flavor Baijiu fermentation process), and tail distillate (the raw spirit collected from the final stages of Baijiu distillation) [12,13,14] (Figure S1). However, despite its wide application and proven effectiveness in industrial production, the intricate microbial function and dynamic evolution of flavor compounds underlying the “double-round bottom fermentation” process remain to be fully elucidated.

With the rapid development of microbiological technologies, particularly high-throughput sequencing and multi-omics approaches, the understanding of microbial community structure and function in Baijiu fermentation has greatly expanded [4,5]. Numerous studies have revealed that lactic acid bacteria, *Bacillus* spp., *Clostridium* spp., yeasts, filamentous fungi and other microbiota are widely found microorganisms in Baijiu fermentation [15,16]. These microorganisms interact within a complex ecological network, participating jointly in raw material degradation and the biosynthesis of alcohols, esters, and organic acids that contribute to the characteristic flavor of Baijiu [16,17,18,19]. However, these studies have primarily focused on community composition, successional dynamics, and microbe-flavor correlations, while the specific mechanisms by which these microorganisms function during the unique “double-round bottom fermentation” process remain poorly understood. In particular, the specific metabolic networks of microbial communities and their associations with the formation of major flavor compounds.

Therefore, this study aims to investigate the microbial community dynamics, metabolic networks, and their influence on major flavor compounds formation during the “double-round bottom fermentation” of strong-flavor Baijiu. By integrating metagenomic sequencing, metabolomic profiling, and physicochemical analyses, this work seeks to elucidate the intrinsic relationships between microbial ecology and flavor formation, thereby providing a scientific basis for process optimization and quality improvement in strong-flavor Baijiu production.

## 2. Materialsand Methods

### 2.1. Sample Collection

Fermented grains (also called Jiupei in Chinese) samples were collected from a representative strong-flavor Baijiu company located in Luzhou, Sichuan province, China. The fermentation pits (also called Jiaochi in Chinese) which had been in continuous use for several decades for traditional fermentation, were selected for this experiment. At the beginning of the DRBF experiment, the pit had just completed a regular 40-day fermentation cycle. After distillation of the upper layers fermented grains, only the compacted bottom layer of fermented grains (approximately 1.0 t) remained in the pit (Figure S1). To initiate DRBF, 18 kg of Daqu, 42 kg of tail distillate, and 42 kg of yellow water were evenly added to this residual bottom layer and thoroughly mixed. Immediately after mixing, samples were collected and designated as Day-0 fermented grain samples, and the height of the fermented grain mass in the pit was measured and recorded to ensure consistent sampling positions on subsequent days (Figure S2). After completion of the DRBF stage described above, the fermented grains were distilled and cooled, and then blended with fresh Daqu and returned to the pit for the next fermentation cycle.

Sampling was performed with a hollow cylindrical sampler [20]. At the fermentation pits, fermented grain was collected from the bottom positions of fermentation pit (approximately 20 cm). Sampling was conducted at 0 (the start of the fermentation process), 2, 4, 6, 8, 10, 15, 20, 30, and 40 days (the end of the fermentation process) (Figure S1). At each time point, three replicate samples (approximately 100 g each) were collected, pooled in sterile plastic bags, and transported in a chilled container at −80 °C for storage until further analysis.

### 2.2. Determination of Physicochemical Factors

The temperature and pH of these samples were measured using a digital thermometer and a pH meter, respectively. The physicochemical properties, such as moisture content, acidity, starch content and reducing sugars, were measured according to the methods specified in the standard T/CBJ 004-2018 [21]. For moisture content, approximately 5 g of well-mixed sample was accurately weighed and dried in a forced-air oven at 105 °C until a constant weight was achieved. Acidity was determined by acid–base titration: 10 g of sample was mixed with water and filtered, and 10 mL of the filtrate was titrated with 0.1 mol/L NaOH to the phenolphthalein endpoint. Starch and reducing sugars contents were quantified using the Fehling reagent method with methylene blue indicator. All determinations were carried out in triplicate [22].

### 2.3. Analysis of Volatile Compounds

Volatile compounds were determined by headspace solid-phase microextraction coupled with gas chromatography–mass spectrometry (HS-SPME-GC-MS) [23]. Approximately 0.5 g of each sample was transferred to a headspace vial, and 8 mL of saturated NaCl solution and 10 μL of 2-octanol (0.728 mg/mL) were added as the internal standard. Subsequent extraction, separation, and identification followed the method of Wang et al. [24]. Volatiles were preconcentrated via HS-SPME using a preconditioned 50/30 μm DVB/CAR/PDMS fiber (Supelco, Bellefonte, PA, USA), The fiber was exposed to the sample headspace at 60 °C for 45 min to achieve adsorption equilibrium. Separation and identification were performed on a Shimadzu QP2020 GC-MS system (Shimadzu, Kyoto, Japan) equipped with a DB-WAX capillary column (60 m × 0.25 mm i.d., 0.25 μm film thickness; J&W Scientific, Folsom, CA, USA). Helium was used as the carrier gas at a constant flow of 1.0 mL/min. The oven temperature followed a programmed gradient: initial hold at 40 °C for 5 min, ramp to 100 °C at 5 °C/min, further ramp to 230 °C at 6 °C/min, and final hold at 230 °C for 10 min. The MS operated in electron impact (EI) ionization mode at 70 eV, full-scan mode from *m*/*z* 40 to 500. The GC-MS interface and ion source were maintained at 250 °C and 230 °C, respectively.

Putative identifications were achieved by matching acquired mass spectra against the NIST-14.0 library, and only matches with similarity indices greater than 80% were accepted. The semi-quantification of volatile compounds was performed using 2-octanol as the internal standard, based on the following formula:

C_2_ = (C_1_ × A_2_)/(A_1_ × m)

where C_2_ is the relative concentration of the volatile compound (μg/g of fermented grains); C_1_ is the concentration of the internal standard (0.728 mg/mL); A_2_ is the peak area of the volatile compound in sample; A_1_ is the peak area of the internal standard; and m is the mass of the fermented grain sample (g).

### 2.4. DNA Extraction

Based on previous study [25], fermented grain (approximately 30 g) was homogenized in 120 mL of sterile phosphate-buffered saline (PBS, pH 8.0). The mixture was then filtered through sterile gauze, and the filtrate was collected. The filtrate was centrifuged (10,000× *g*, 10 min) to pellet the microbial cells. The resulting cell pellet was washed three times with PBS. Each washing step consisted of vortexing for 10 min, followed by centrifugation (10,000× *g*, 5 min) to collect the microbial cells. The final cell pellet was transferred to a 50 mL centrifuge tube and resuspended in 10 mL of DNA extraction buffer (0.1 M Tris-HCl, 0.1 M NaCl, 1% CTAB, pH 8.0). For enzymatic lysis, the snailase and lysozyme were added to the suspension and incubated at 37 °C for 2.5 h, achieving final concentrations of 4 mg/mL and 3 mg/mL, respectively. Subsequently, 200 µL of 20% SDS was added, and the mixture was incubated at 65 °C for 1.5 h. Protein digestion was initiated by adding proteinase K (the final concentration was 200 μg/mL) to the lysate, followed by incubation for 1 h at 55 °C in a shaking water bath. After incubation, the sample was centrifuged (5000× *g*, 4 min), and the supernatant was collected. The supernatant was then purified via two sequential extractions. First, an equal volume of phenol:chloroform:isoamyl alcohol (25:24:1, *v*/*v*/*v*) was added, and the mixture was centrifuged (10,000× *g*, 10 min). The upper aqueous phase was transferred to a new tube, and this step was repeated once. Next, an equal volume of chloroform:isoamyl alcohol (24:1, *v*/*v*) was added, followed by centrifugation (10,000× *g*, 10 min). The resulting aqueous phase was collected, and this extraction step was repeated once. To precipitate the DNA, 0.6 volumes of ice-cold isopropanol were added to the final aqueous phase, and the solution was incubated at −20 °C for 2 h. The precipitated DNA was collected by centrifugation (13,000× *g*, 10 min), and then the DNA pellet was washed with ice-cold 70% (*v*/*v*) ethanol and centrifuged again (13,000× *g*, 10 min). Finally, the dried DNA pellet was dissolved in 100 μL of sterile Tris-EDTA buffer (pH 8.0), aliquoted, and stored at −80 °C for subsequent sequencing.

### 2.5. Processing of Sequencing Data

The amplicons and metagenomic sequencing were performed by Meiji Biotech Co., Ltd. (Shanghai, China). Primers for bacterial 16S rRNA gene (V3-V4 region) and fungal ITS rRNA gene (ITS1-ITS2 region) sequencing were selected based on a previous report [22]. Raw sequence data were processed using the Quantitative Insights Into Microbial Ecology (QIIME, version 1.9.1) pipeline. Sequences were quality-filtered as described by Xiao et al. [24]. Subsequent clustering of high-quality sequences into Operational Taxonomic Units (OTUs) was performed at a 97% similarity threshold using USEARCH (version 10.0.240). Taxonomic classification of the OTUs was performed against the Greengenes database (for bacteria) and the UNITE database (for fungi) to determine the community composition at genus taxonomic levels.

To elucidate the functional shifts within the microbial community during fermentation, a metagenomic analysis was conducted on the fermented grain samples. The data processing workflow was adapted from the method described by Bai et al., with minor modifications [26]. The raw sequence data were quality-controlled using fastp (version 0.23.2) with default settings. And then quality-controlled reads followed by KneadData (version 0.12.0) to isolate high-quality microbial reads, with plants reads mapped to sorghum (https://www.ncbi.nlm.nih.gov/datasets/genome/GCF_000003195.3/, accessed on 31 March 2025) and wheat (https://www.ncbi.nlm.nih.gov/datasets/genome/GCF_018294505.1/, accessed on 31 March 2025) reference genome and removed using Bowtie2 (version 2.5.1). The remaining quality-filtered reads were assembled using MEGAHIT (version 1.2.9). Prodigal (version 2.6.3) was used for gene prediction, and assembled contigs were clustered using CD-HIT (version 4.8.1) with 95% identity and 90% coverage. Then Salmon (version 1.8.0) was employed to measure the abundance of contigs in each sample. Protein functions, including KEGG Ortholog (KO) were annotated using eggNOG-mapper based on eggNOG (version 5.0) clusters.

### 2.6. Statistical Analysis and Visualization

The mean ± SD data for parallel samples are reported. Principal coordinate analysis (PCoA) based on Bray–Curtis dissimilarity was employed to assess the similarity of microbial communities and volatile metabolite profiles across samples, using the Majorbio Cloud platform (https://cloud.majorbio.com, accessed on 24 July 2025). Figures were generated using the R software (version 4.3.1) and Origin 2024 (OriginLab, Northampton, MA, USA).

## 3. Results and Discussion

### 3.1. Variation in Physicochemical Parameters During DRBF

**Figure 1:**
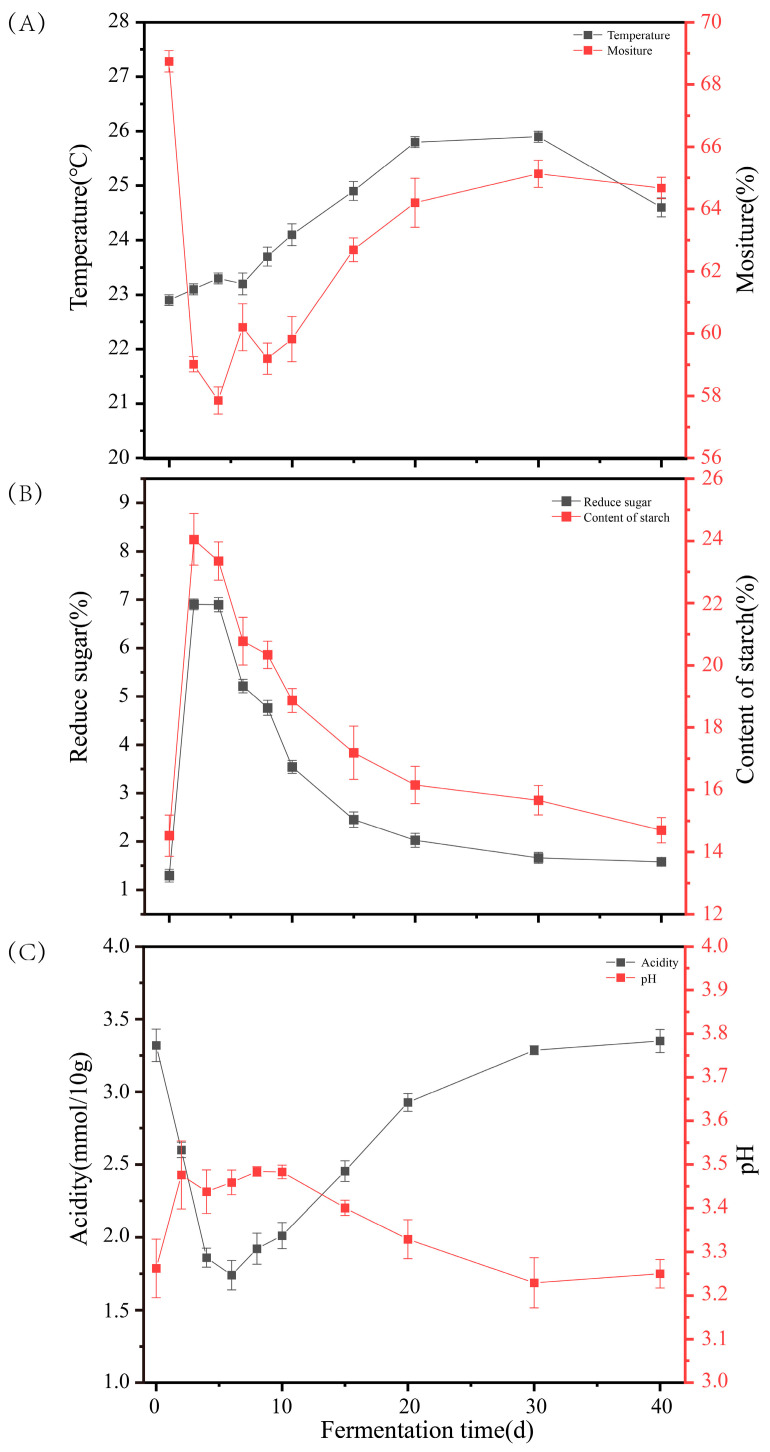
Changes in physicochemical parameters during the DRBF process. (**A**) Temperature and moisture; (**B**) Reducing sugars and starch; (**C**) Acidity and pH.

During the DRBF process, the temperature of the fermented grains showed a gradual and steady increase, rising from an initial 22.9 ± 0.10 °C at cellar entry to 24.6 ± 0.17 °C at the end of fermentation (Figure 1A). During Days 6–30, the temperature remained elevated, culminating in a maximum of 25.9 ± 0.10 °C on Day 30. This temperature profile was consistent with the findings of Cheng et al. [27], where the peak temperature was typically lower and occurred later compared to traditional single-round strong-flavor fermentation processes [28].

Moisture content exhibited a biphasic pattern. It was 68.74 ± 0.34% on Day 0, declined sharply during the early phase, and then gradually increased after Day 6, stabilizing at 62.69–65.13% during Days 15–40 (Figure 1A).

The reducing sugar and starch contents were initially lower than those observed in traditional fermentation, primarily due to the absence of new sorghum material [29,30] (Figure S1). They increased rapidly during the first two days, peaking at 6.90 ± 0.11% and 24.05 ± 0.83%, respectively, likely as a result of the downward migration of substrates from the upper layers. Thereafter, their concentrations decreased steadily as a consequence of microbial metabolism, reaching 1.58 ± 0.07% and 14.70 ± 0.41% by the end of fermentation (Figure 1B).

Acidity decreased from 3.32 ± 0.11 on Day 0 to 1.74 ± 0.10 on Day 6, and then gradually increased to 3.35 ± 0.08 at the end of fermentation. Meanwhile, the pH fluctuated between 3.22 and 3.48. This stability was likely attributed to the buffering capacity of the fermentation matrix and the dynamic balance between acid production (e.g., lactic and acetic acids) and consumption (Figure 1C).

In the present DRBF system, the starting substrate differed markedly from that of a traditional fermentation. The initial fermented grains mainly originated from the bottom layer of the previous standard 40-day cycle and therefore already contained relatively abundant flavor compounds, together with elevated moisture and acidity but reduced starch and reducing sugar contents [19,28]. In addition, defined amounts of Daqu, yellow water and tail distillate were added in this experiment, corresponding to approximately 2%, 4% and 4% of the total mass of fermented grains, respectively. These additions further increased the moisture content, acidity and volatile load of the day-0 substrate. Moreover, because this material was located at the bottom of the pit, it was subjected to strong gravitational pressure and liquid migration from the upper fermented grains (Figure S2). Under such conditions, rapid loss of free water and soluble components from the bottom layer would be expected at the beginning of DRBF. Consistent with this substrate condition, sharp decreases were observed in moisture, reducing sugars and volatile compounds from Day 0 to Day 2.

### 3.2. Variation in Volatile Compounds During DRBF

**Figure 2:**
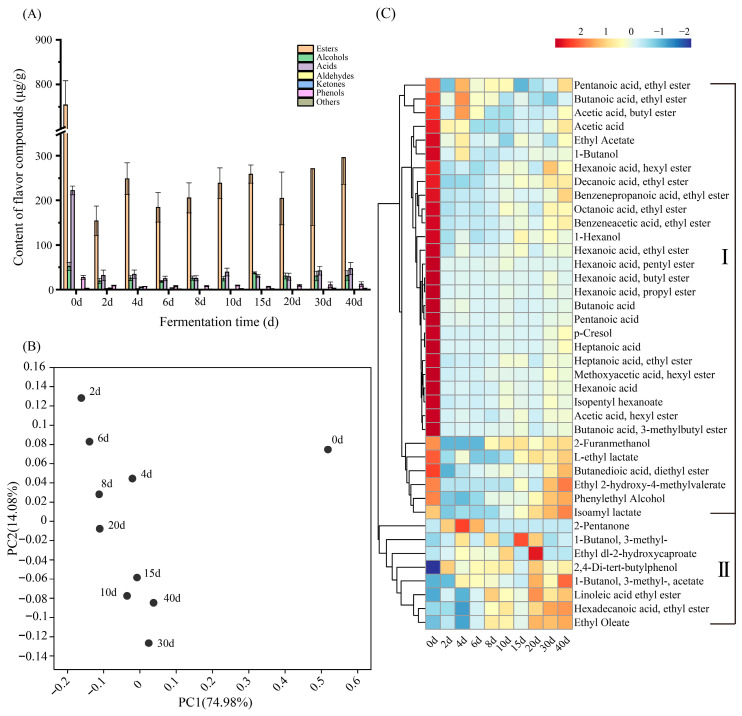
The variation in volatile compounds during the DRBF process. (**A**) Types and concentration of volatile compounds; (**B**) PCoA of the volatile compounds based on concentration within fermented grains; (**C**) Heatmap of top 40 volatile compounds in different time from process, color scale represents z score-normalized abundance.

A total of 153 volatile compounds were identified in the fermented grains during DRBF, including 76 esters, 23 alcohols, 15 acids, 4 aldehydes, 9 ketones, 10 aromatic compounds, and 17 compounds classified as “others”. Esters, alcohols, acids, and aromatic compounds were the predominant classes in the volatile profile of the fermented grains (Figure 2A). On Day 0, their concentrations were 753.85 ± 54.13 μg/g, 51.87 ± 9.00 μg/g, 221.81 ± 9.72 μg/g, and 27.36 ± 4.27 μg/g, respectively. By Day 2, they decreased sharply to 153.97 ± 33.15 μg/g, 19.41 ± 5.69 μg/g, 31.71 ± 11.96 μg/g, and 9.01 ± 0.95 μg/g, before gradually increasing to 295.81 ± 60.12 μg/g, 31.60 ± 10.73 μg/g, 46.95 ± 13.30 μg/g, and 12.42 ± 4.96 μg/g at the end of fermentation.

The volatile composition of DRBF showed an initially high content followed by a significant decline, which could be attributed to the use of fermented grains from the previous batch as the initial substrate, as well as the addition of Daqu, yellow water [11] and tail distillate [12]. These practices contributed to elevated baseline levels of volatile compounds on Day 0. Moreover, since the fermented grains were located at the bottom of the pit, gravitational pressure from upper layers likely led to the loss of volatiles along with moisture, explaining the sharp early decline. As fermentation progressed, microbial activity promoted the synthesis and accumulation of esters, alcohols, and acids, resulting in a gradual increase in total volatile content during the mid-to-late stages (10–40 d) [31]. Principal Coordinates Analysis (PCoA) revealed that the volatile profiles between Days 10 and 40 were highly similar, indicating the formation of a stable aroma structure (Figure 2B).

To elucidate the dynamics of key aroma components, the 40 most abundant volatile compounds—including 27 esters, 5 alcohols, 5 acids, 1 ketone, and 2 aromatic compounds—were selected for further analysis (Figure 2C). These compounds were classified into two distinct patterns. The first group comprised compounds with high initial concentrations that decreased early and increased again after Day 10, such as butanoic acid, acetic acid, hexanoic acid, ethyl acetate, ethyl hexanoate, and L-ethyl lactate. The second group included compounds with low initial concentrations that increased steadily throughout fermentation, including ethyl linoleate, ethyl palmitate, and ethyl oleate.

### 3.3. Diversity of Microbial Communities During DRBF

**Figure 3:**
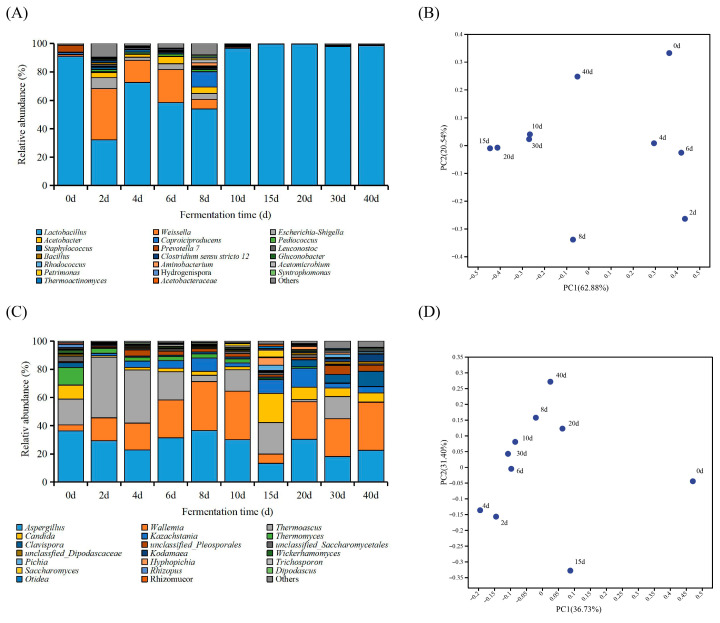
The composition of abundant microorganisms at genus level during the DRBF process. (**A**) Genera of bacterial communities with relative abundance >0.1%; (**B**) PCoA of bacterial communities within fermented grains; (**C**) Genera of fungal communities with relative abundance >0.3%; (**D**) PCoA of fungal communities within fermented grains.

High-throughput sequencing identified 505 bacterial genera during DRBF. The bacterial community was dominated by 20 genera (>0.1% relative abundance) (Figure 3A), which collectively accounted for over 90.7% of total reads, including *Lactobacillus*, *Weissella*, and *Bacillus*—genera commonly associated with Baijiu fermentation [28,32]. At the start of fermentation, *Lactobacillus* was the most abundant genus, with a relative abundance of 90.89%. Between Days 2 and 8, a significant decline in *Lactobacillus* abundance was observed, with an average relative abundance of 54.31%. During this period, the relative abundances of *Weissella*, *Escherichia-Shigella*, *Acetobacter*, *Caproiciproducens*, *Pediococcus*, and *Staphylococcus* increased markedly, reaching average relative abundances of 20.36%, 4.53%, 3.85%, 2.90%, 1.53%, and 1.28%, respectively. After Day 10, *Lactobacillus* emerged as the predominant bacterial genus once more, with an average relative abundance of 98.41%, which was maintained throughout the remainder of the fermentation process, whereas other genera remained below 0.3%. Principal Coordinates Analysis (PCoA) revealed that the bacterial community structure stabilized after Day 10 (Figure 3B).

A total of 175 fungal genera were identified, with 20 genera (>0.3% relative abundance) accounting for 94.87% of total fungal reads (Figure 3C). The dominant fungal genera included *Aspergillus*, *Wallemia*, and *Thermoascus*. At the start of fermentation, the fungal community was primarily composed of *Aspergillus*, *Thermoascus*, *Candida*, and *Thermomyces*, with relative abundances exceeding 10%, most likely originating from Daqu and raw materials. By Day 2, the combined relative abundances of *Aspergillus*, *Thermoascus*, and *Wallemia* reached 88.59% and remained dominant throughout fermentation, whereas *Thermomyces* exhibited a sharp decrease from 12.39% on Day 0 to below 0.2% at the end. The yeasts *Candida* and *Kazachstania*—both key genera in Baijiu fermentation [33]—showed contrasting trends: the relative abundance of *Candida* declined rapidly from 10.0% on Day 0 to 2.74% on Day 8, whereas *Kazachstania* increased from 0.09% to 9.44% over the same period. After Day 8, both genera increased again, with average relative abundances of 8.81% and 6.74%, respectively. Other yeast genera decreased gradually at the beginning of fermentation, followed by stabilization. Notably, *Clavispora* decreased to 1.0% on Day 15, but subsequently rose to 10.8% by the end of fermentation, likely due to its ethanol tolerance [34]. PCoA indicated that, except for Day 15, the fungal community structure stabilized after Day 6 of fermentation (Figure 3D).

### 3.4. Function Profile During DRBF

**Figure 4:**
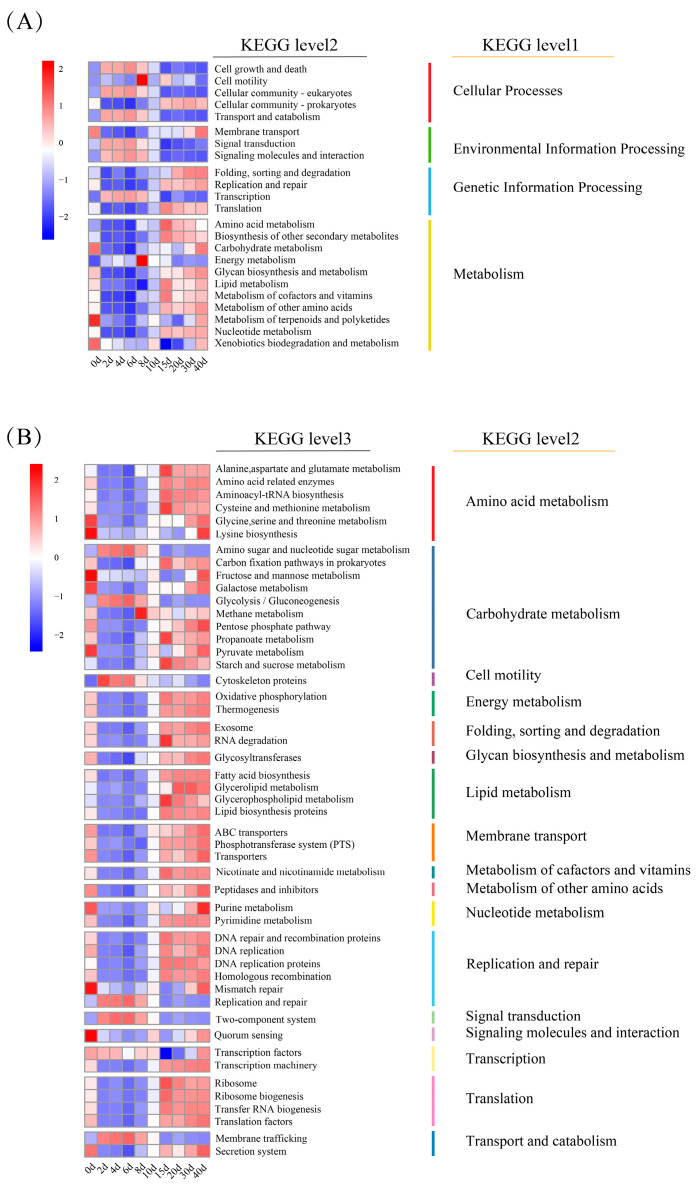
KEGG-based functional annotation of genes revealed shifts in functional profiles during the DRBF process. (**A**) Distribution of KEGG functional categories at levels 1 and 2; (**B**) Top 50 KEGG level 3 functions; color scale represents z score-normalized abundance.

Functional annotation of microbial genes identified four major KEGG functional categories: Metabolism, Genetic Information Processing, Environmental Information Processing, and Cellular Processes. Metabolic activity exhibited a clear time-dependent pattern (Figure 4A), which was similar to the findings of Lin et al. [31].

During the early stage (0–8 d), pathways related to rapid microbial proliferation—such as Cell Growth and Death, Cellular Community–Eukaryotes, Transport and Catabolism, Signal Transduction, and Energy Metabolism—were highly represented. In the mid-to-late stage (10–40 d), functional activity shifted toward pathways associated with complex flavor biosynthesis and environmental stress adaptation (e.g., low pH and osmotic pressure). Correspondingly, pathways involved in the biosynthesis of other secondary metabolites, amino acid metabolism, and protein folding, sorting, and degradation showed elevated relative abundances.

To further elucidate functional dynamics, the top 50 level-3 KEGG pathways were analyzed, most of which were primarily associated with metabolism and genetic information processing. At the early stage (0–8 d), pathways such as glycolysis/gluconeogenesis, cytoskeleton proteins, and replication and repair were predominant (Figure 4B). As fermentation progressed, pathways related to flavor compound biosynthesis and stress response remained relatively abundant. These included amino acid metabolism-related enzymes, glycosyltransferases, fatty acid biosynthesis, and glycerolipid metabolism. Additionally, DNA repair pathways, such as mismatch repair and homologous recombination, remained highly abundance throughout the mid-to-late stages (10–40 d) (Figure 4B).

### 3.5. Reconstruction of Metabolic Pathways and Key Enzyme in Substrate Degradation and Flavor Formation During DRBF

**Figure 5:**
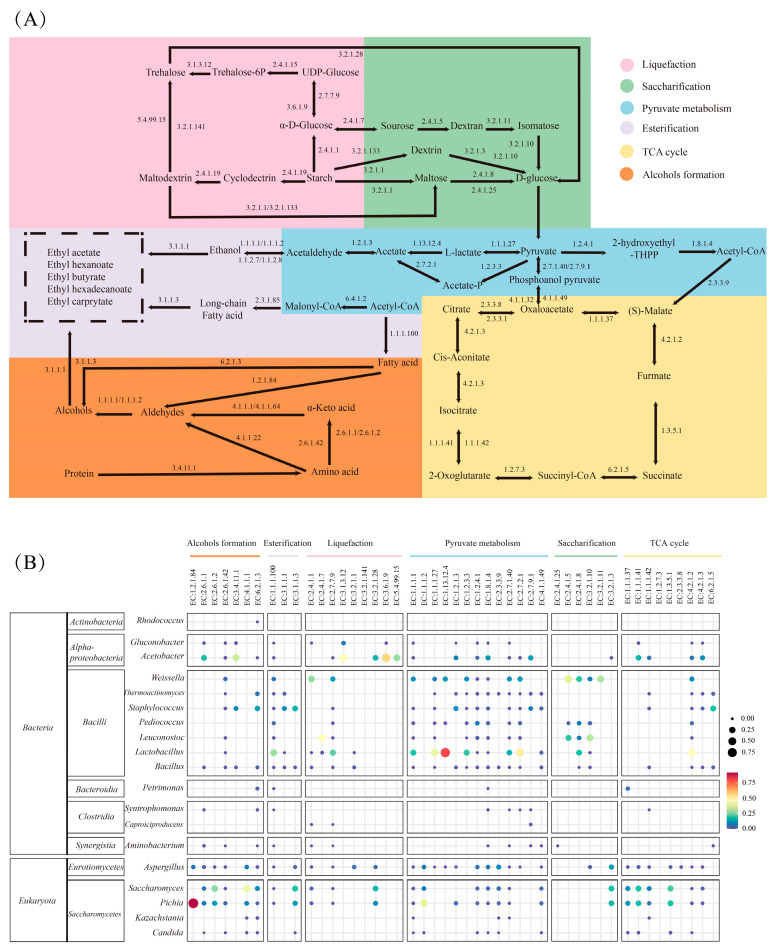
Reconstruction of metabolic pathways and key enzyme abundances related to substrate degradation and flavor formation based on KEGG annotations. (**A**) Metabolic pathways reconstructed from metagenomic datasets; (**B**) Distribution of enzymes among microbial genera matched from the metagenomic data.

To elucidate the role of microbial communities in substrate degradation and flavor compound formation during DRBF, metabolic pathways were reconstructed from metagenomic data, and the abundances of 57 key enzymes involved in these pathways were profiled (Figure 5A).

In the reconstructed liquefaction and saccharification pathways (Figure 5A), UDP-glucose pyrophosphorylase (EC: 2.7.7.9) and sucrase–isomaltase (EC: 3.2.1.10) remained highly abundant throughout fermentation, whereas cyclomaltodextrin glucanotransferase (EC: 2.4.1.19) and glucoamylase (EC: 3.2.1.3) persisted at relatively low levels. Other saccharolytic enzymes showed elevated levels during the early stage (0–8 d) before declining during the mid-to-late stage (10–40 d) (Figure S3). Correlation analyses with dominant microbial genera (relative abundance > 0.01%) revealed that glycogen phosphorylase (EC: 2.4.1.1), α-amylase (EC: 3.2.1.1), maltose phosphorylase (EC: 2.4.1.8), and amylomaltase (EC: 2.4.1.25) were broadly distributed among *Acetobacter*, *Weissella*, *Leuconostoc*, *Lactobacillus*, *Aspergillus*, *Saccharomyces*, and *Pichia* (Figure 5B).

Pyruvate metabolism functioned as a central metabolic hub, linking carbohydrate degradation with the synthesis of flavor precursors [29]. Methanol dehydrogenase (cytochrome c) (EC: 1.1.2.7) and acetyl-CoA carboxylase (EC: 6.4.1.2) remained at low abundance throughout fermentation, whereas pyruvate dehydrogenase (EC: 1.2.4.1), pyruvate oxidase (EC: 1.2.3.3), acetate kinase (EC: 2.7.2.1), L-lactate dehydrogenase (EC: 1.1.1.27), and multiple alcohol dehydrogenases (EC: 1.1.1.1 and EC: 1.1.1.2) were at high abundance.

The tricarboxylic acid (TCA) cycle, a central pathway in Baijiu fermentation, was reconstructed (Figure 5A). Its associated enzymes showed consistently high abundances throughout fermentation, peaking during the early stage (Figure S3). The TCA cycle not only produced organic acids, including oxalic, citric, and isocitric acids, contributing to the characteristic flavor profile, but also supplied key metabolic intermediates for subsequent biosynthetic pathways [35].

Ester formation, an essential determinant of Baijiu aroma, proceeds via enzymatic esterification of alcohols and acids [36]. In the reconstructed ester and alcohol biosynthesis network (Figure 5A), branched-chain-amino-acid transaminase (EC: 2.6.1.42) and β-ketoacyl reductase (EC: 1.1.1.100) remained abundant, whereas most other enzymes displayed higher abundances during the early stage (0–8 d) relative to the mid-to-late stage (10–40 d). In addition to glycolysis, amino acid catabolism played a key role in higher alcohol formation through transaminases and decarboxylases (e.g., aspartate transaminase EC:2.6.1.1, alanine transaminase EC: 2.6.1.2, and pyruvate decarboxylase EC: 4.1.1.1). Fatty acids were converted into alcohols through the action of fatty acid thiokinase (EC: 6.2.1.3), alcohol-forming fatty acyl-CoA reductase (EC: 1.2.1.84), and alcohol dehydrogenases. Finally, alcohols and acids were esterified, yielding key aroma esters via carboxylesterase (EC: 3.1.1.1) and lipase (EC: 3.1.1.3) [37].

In strong-flavor Baijiu, the formation of key flavor compounds, especially alcohols and esters, is supported by a set of core enzymes that occupy central positions in the main carbon and flavor-forming pathways. Metagenomic analysis showed that pyruvate dehydrogenase (EC: 1.2.4.1), alcohol dehydrogenase (EC: 1.1.1.1 and EC: 1.1.1.2), and lipase/esterase (EC: 3.1.1.3 and EC: 3.1.1.1) were persistently abundant during the DRBF process and were encoded by multiple dominant genera (Figure 5 and Figure S3). In the simplified metabolic scheme (Figure S4), starch in the fermented grains was first hydrolyzed to D-glucose mainly by *Aspergillus* (e.g., EC: 3.2.1.1, EC: 3.2.1.3, EC: 3.2.1.10). The resulting glucose then entered the glycolytic pathway and was converted to pyruvate, which was further transformed into lactate and acetate, with *Lactobacillus* and *Aspergillus* contributing key steps in these routes (e.g., EC: 1.1.1.27, EC: 1.2.3.3, EC: 1.13.12.4, EC: 2.7.2.1). Pyruvate dehydrogenase, mainly encoded by *Acetobacter*, *Lactobacillus*, *Aspergillus*, *Saccharomyces*, and *Pichia* (EC: 1.2.4.1, EC: 1.8.1.4), formed a major metabolic hub by converting pyruvate to acetyl-CoA and thereby sustaining a relatively stable acetyl-CoA pool for the tricarboxylic acid cycle, fatty-acid biosynthesis, and downstream ester formation. Alcohol dehydrogenases were likewise widely encoded in these genera and in other bacteria such as *Weissella*, *Leuconostoc*, and *Bacillus* (e.g., EC: 1.1.1.1, EC: 1.1.1.100). These observations are consistent with other solid-state fermentations, where pyruvate-utilizing enzymes and acyl-CoA metabolism genes are enriched in flavor-forming microbial community [38]. This broad taxonomic distribution meant that both sugar-derived and amino-acid-derived α-ketoacids could be continuously reduced to higher alcohols throughout the fermentation. In addition, amino-acid transamination to α-ketoacids (EC: 2.6.1.42, EC: 2.6.1.1, EC: 2.6.1.2) was catalyzed by the same dominant genera, while molds and yeasts, especially *Aspergillus*, *Saccharomyces*, and *Pichia*, further oxidized these intermediates to aldehydes (e.g., EC: 1.2.1.84, EC: 4.1.1.1), which were then reduced by alcohol dehydrogenases to fusel alcohols. In addition, amino-acid transamination to α-ketoacids (EC: 2.6.1.42, EC: 2.6.1.1, EC: 2.6.1.2) was catalyzed by the same dominant genera, while molds and yeasts, especially *Aspergillus*, *Saccharomyces*, and *Pichia*, further oxidized these intermediates to aldehydes (e.g., EC: 1.2.1.84, EC: 4.1.1.1), which were then reduced by alcohol dehydrogenases to alcohols. Finally, lipase (EC: 3.1.1.3) derived mainly from molds and yeasts, together with esterase from *Lactobacillus* (EC: 3.1.1.1), catalyzed the condensation of organic acids with these alcohols and promoted the formation of a wide spectrum of esters. Overall, the persistent high relative abundance and multi-taxa distribution of pyruvate dehydrogenases, alcohol dehydrogenases, and lipase/esterase ensured that the key nodes of acetyl-CoA supply, alcohol formation, and esterification remained active throughout the DRBF process.

## 4. Conclusions

This study provides comprehensive insights into the microbial ecology and functional metabolism underlying DRBF in strong-flavor Baijiu production. By combining physicochemical monitoring, volatile profiling, and metagenomic analyses, we delineated the dynamic and coordinated relationships among physicochemical transformations, microbiota succession, and metabolic functions across the fermentation process. The results indicate that microbial succession, synergistic enzymatic activities, and metabolic regulation jointly drive substrate degradation and aroma biosynthesis through interconnected carbohydrate utilization, pyruvate metabolism, the tricarboxylic acid (TCA) cycle, and esterification pathways. Bacteria, yeasts, and molds exhibited cooperative metabolic functions that sustained the continuous conversion of starch-derived substrates into aroma-active compounds. The persistent abundance of key enzymes, including pyruvate dehydrogenase, alcohol dehydrogenase, and lipase, further underscores their crucial contributions to alcohol and ester formation. Collectively, the reconstructed metabolic networks highlight a multi-taxon cooperative system that ensures the continuity of fermentation and the development of characteristic flavor. Overall, these findings advance the understanding of microbial cooperation and metabolic regulation in DRBF and provide a theoretical foundation for optimizing fermentation control and improving flavor quality in Baijiu manufacturing.

## Data Availability

The original contributions presented in the study are included in the article/Supplementary Materials. Further inquiries can be directed to the corresponding authors.

## Supplementary Materials

The following supporting information can be downloaded at: https://www.mdpi.com/article/10.3390/foods14244228/s1, Figure S1: Process flow diagram of double-round bottom fermentation (DRBF) of strong-flavor Baijiu; Figure S2: Cross-sectional schematic of the fermentation pit used for DRBF; Figure S3: Temporal dynamics of key enzyme abundances during DRBF; Figure S4: Distribution of enzymes in five microorganisms to key metabolic pathways.
