# Supplementary material for: Multi-Omics Insights into Microbial Community Dynamics and Functional Shifts During Double-Round Bottom Fermentation of Strong-Flavor Baijiu
Source: Foods. 2025 Dec 9;14(24):4228. doi: 10.3390/foods14244228 (PMC12732256; doi:10.3390/foods14244228)
Supplement: Supplementary file 1 [file foods-14-04228-s001.zip › foods-4002702-supplementary.pdf]

Supplementary file

Multi-omics insights into microbial community dynamics and functional shifts during double-round bottom fermentation of strong-flavor Baijiu

Jiao Li [1,2], Yaqi Guo [1,2], Yang Yang [3,4], Shu Li [3,4], Tao Xu [3,4], Ruiqi Zeng [1,2], Songtao Wang [3,4], Caihong Shen [3,4], Zhenghong Xu [5], Yong Zuo [1,2*], Chen Xiao [1,2*]

[1] Key Laboratory of the Evaluation and Monitoring of Southwest Land Resources (Ministry of Education), Sichuan Normal University, Chengdu, Sichuan, China

[2] College of Life Science, Sichuan Normal University, Chengdu, Sichuan, China

[3] Luzhou Pinchuang Technology Co., Ltd/ National Engineering Research Center of Solid-State Brewing, Luzhou, Sichuan, China

[4] Luzhou Laojiao Co. Ltd., Luzhou, Sichuan, China

[5] Innovation Center for Advanced Brewing Science and Technology, Sichuan University, Chengdu, China

[*]Corresponding author: Yong Zuo, Chen Xiao

Email address: skzuoyong@sicnu.edu.cn, xiaochen_2022@sicnu.edu.cn

Supplementary Figure 1

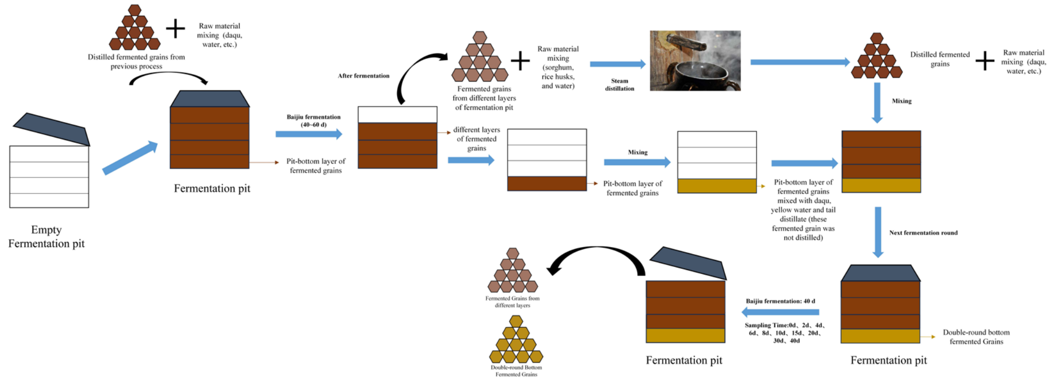

Figure S1. Process flow diagram of double-round bottom fermentation (DRBF) for strong-flavor Baijiu.

Supplementary Figure 2

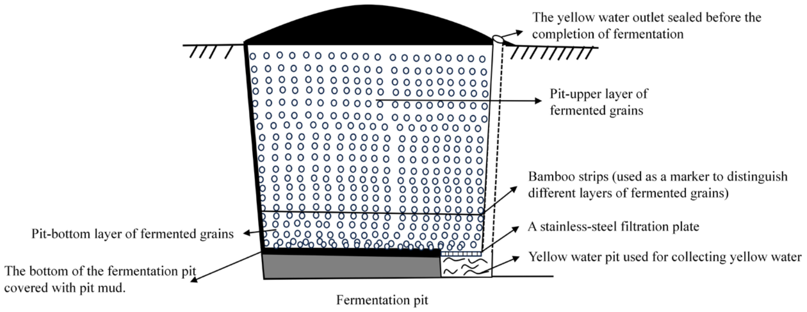

Figure S2. Cross-sectional schematic of the fermentation pit used for DRBF.

Supplementary Figure 3

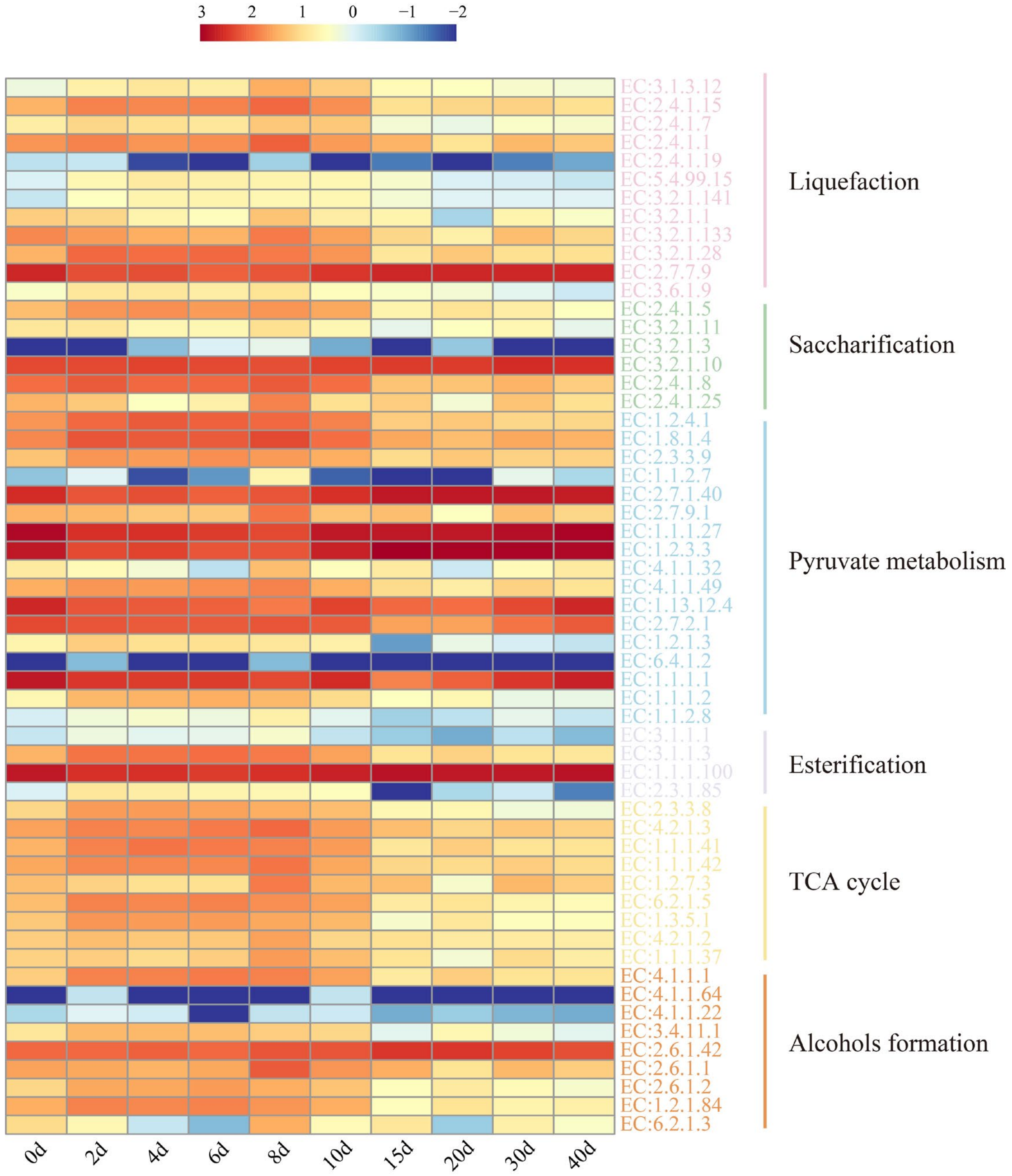

Figure S3. Temporal dynamics of key enzyme abundances during DRBF,the color gradient indicated the abundance variation z-value normalized values.

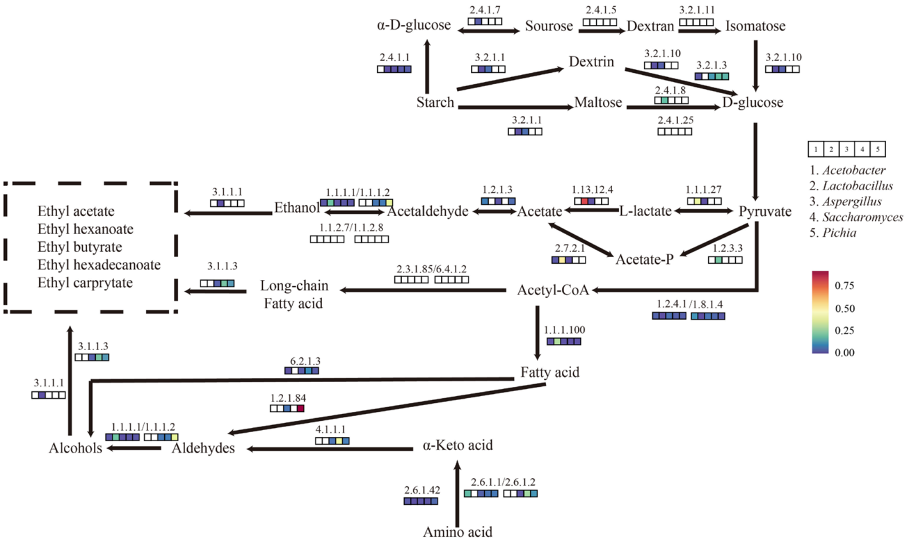

Figure S4. Distribution of enzymes in five microorganisms to key metabolic pathways.

Note: The numbers in the legend correspond to the five microorganisms (1–5) as listed: 1. *Acetobacter*, 2. *Lactobacillus*, 3. *Aspergillus*, 4. *Saccharomyces*, 5. *Pichia*. The heatmap squares represent the abundances of each microorganism to enzyme, with the organisms corresponding to the numbers 1 through 5 as listed in the legend.
